# SECTM1 acts as an immune-related biomarker of poor prognosis and promotes cancer progression by modulating M2 macrophage polarization in esophageal squamous cell carcinoma

**DOI:** 10.3389/fimmu.2025.1507227

**Published:** 2025-01-29

**Authors:** Pengzhou Kong, Ye Jiao, Meng Sun, Zhinan Zhou, Yingying Zhang, Xin Yang, Jing Ren, Mengyuan Yang, Yanyan Dong, Bin Song

**Affiliations:** ^1^ Translational Medicine Research Center and Department of Pathology, Shanxi Medical University, Taiyuan, Shanxi, China; ^2^ Key Laboratory of Cellular Physiology (Shanxi Medical University), Ministry of Education, Department of Pathology, Shanxi Medical University, Taiyuan, Shanxi, China; ^3^ State Key Laboratory for Pneumoconiosis of National Health Commission, Key Laboratory of Prevention, Treatment and Fundamental Studies for Respiratory Diseases of Shanxi, Department of Respiratory and Critical Care Medicine, First Hospital of Shanxi Medical University, Taiyuan, Shanxi, China; ^4^ Department of Pathology, Shanxi Provincial People’s Hospital, The Fifth Clinical Medical College of Shanxi Medical University, Taiyuan, China; ^5^ Cancer Center, Shanxi Bethune Hospital, Shanxi Academy of Medical Sciences, Tongji Shanxi Hospital, Third Hospital of Shanxi Medical University, Taiyuan, Shanxi, China

**Keywords:** ESCC, SECTM1, immune, biomarker, macrophage, CCL5

## Abstract

Esophageal squamous cell carcinoma (ESCC) is the most prevalent primary malignant esophageal tumor in China and has a poor prognosis, but lacks effective diagnostic and prognostic biomarkers. Through single-sample gene set enrichment analysis (ssGSEA), we conducted immune genomic analysis based on 28 immune features using transcriptomic data from 155 ESCC cases. We established of two ESCC subtypes characterized by high and low immune profiles, and 352 differentially expressed immune genes were identified between the two subtypes. Performed with univariate and multivariate Cox regression, a novel prognostic prediction model was developed based on three immune-related genes (MAP3K8, SECTM1, IGLV7-43), which has been identified as a relatively accurate, independent, and specific prognostic risk model for ESCC patients in different ESCC cohorts. Furthermore, SECTM1 was upregulated in ESCC tissues and associated with adverse clinical outcomes. In cell experiments, overexpression of SECTM1 effectively promoted the proliferation, migration, and invasion of ESCC cells, while SECTM1 knockdown significantly inhibited these cellular processes. Furthermore, its overexpression promoted macrophage polarization towards the M2-like phenotype and promoted the migration of M2-like macrophage cells and C-C Motif Chemokine Ligand 5 (CCL5) was the key mediator in the pro-cancer effect of SECTM1. In a Conclusion, our study established a prognostic prediction model based on immune-related gene signature, which provided a reliable prognostic tool for ESCC and identified SECTM1 as a potential biomarker in ESCC.

## Introduction

Esophageal cancer ranks as the eighth most common cancer globally (1). Over half of esophageal cancer cases occur in China, with the predominant histological types being Esophageal Squamous Cell Carcinoma (ESCC), particularly prevalent in regions such as the Taihang Mountains at the junction of Henan, Shanxi, and Hebei, as well as the Fujian-Guangdong area. The lack of early-specific symptoms and the absence of specific tumor markers and effective therapeutic targets lead to late discovery, poor treatment efficacy, and unfavorable clinical prognoses of ESCC, with a 5-year overall survival (OS) rate of only 20% (2). Therefore, there is an urgent need to search for new prognostic and diagnostic biomarkers for ESCC, as well as therapeutic targets.

The initiation and progression of tumors are closely related to their microenvironment (3). The tumor microenvironment (TME) consists of tumor cells, immune and inflammatory cells, tumor-associated fibroblasts, microvessel cells, stromal tissues, and cytokines and chemokines (4). TME significantly influences tumor growth, invasion, and metastasis, with various components interacting to either promote or inhibit tumor development (5). Wherein tumor-associated macrophages (TAMs) are a major immune cell population in the TME, playing a crucial role in the progression of tumors. Based on gene expression characteristics and functions, TAMs are usually categorized into M1 and M2 types, with M1 macrophages possessing anti-tumor functions, while M2 macrophages exhibit pro-tumor activities (6). Cancer immunotherapy has demonstrated significant success in treating various malignancies (7–11). Thus, immune-related genes (IRGs) have enormous potential to become biomarkers for tumor diagnosis and prognosis. However, there is still a lack of relevant research in ESCC.

Secreted And Transmembrane 1 (SECTM1), one transmembrane and secreted protein, is characterized by type 1a transmembrane protein features (12), which expressed on the cell membrane and has been identified as a ligand for CD7 (13). SECTM1 is associated with the activation and regulation of T cells and natural killer (NK) cells (14, 15). Moreover, SECTM1 has been identified as a potential predictive biomarker for the response to immunotherapy in various types of cancer, showing upregulation in immune-hot tumors and sensitivity to the IFN-γ/STAT1 signaling pathway (16). However, the clinical and functional role of SECTM1 in ESCC lack in-depth investigation.

In this study, through the analysis of transcriptomic data from 155 cases of ESCC and The Cancer Genome Atlas Program (TCGA)-ESCC, we constructed a prognostic prediction model for IRGs in ESCC. This model effectively predicts the 1-year, 2-year, and 3-year survival rates of ESCC patients. From the prognostic model, we selected SECTM1 gene for further investigation to its potential pro-cancer effects. Bioinformatics analyses research showed SECTM1 was higher expressed in ESCC tissues compared to adjacent non-cancerous tissues and its elevated expression was associated with poor prognosis and immune cell infiltration. Further functional analyses revealed SECTM1 not only promoted malignant behaviors in ESCC cells but also facilitated the M2 polarization of macrophages, aligning with chemokine signaling pathways, particularly involving C-C Motif Chemokine Ligand 5 (CCL5). All of the results suggested SECTM1 act as a potential biomarker and therapeutic target for ESCC.

## Materials and methods

### Selection of immune-related genes

2, 483 immune-related genes (IRGs) were collected from IMMPORT in October 2022 (https://www.immport.org/home) (17). And IRGs were listed in **Supplementary Table S1**.

### Data collection and preprocessing

We utilized RNA-seq data from 155 cases of ESCC provided by our group (https://pubmed.ncbi.nlm.nih.gov/36584672/). The RNA-seq data of the TCGA ESCC samples were downloaded from the TCGA website (https://portal.gdc.cancer.gov/) for model validation. This dataset comprises a total of 81 samples, with TCGA-V5-A7RC-06A and TCGA-V5-A7RC-01B identified as duplicate samples. In this study, the transcriptomic data for these two samples were averaged, resulting in 80 unique samples for analysis within the TCGA dataset. The expression data and clinical features of 119 ESCC samples (GSE53625) were obtained from the Gene Expression Omnibus (GEO) database (https://www.ncbi.nlm.nih.gov/geo/query/acc.cgi?acc=GSE53625).

### Consensus clustering analysis and construction of molecular subtypes

We analyzed on the transcriptome data of 155 samples using the “GSVA” R package to quantify the enrichment levels of 28 immune signals in each ESCC sample. To investigate the effectiveness of ssGSEA scores in classifying ESCC patients, we performed unsupervised K-Means clustering analysis. Utilizing the “ConsensusClusterPlus” R package with 1000 iterations, we conducted a consensus clustering analysis on ESCC patients based on ssGSEA scores from the 155 samples. We determined the optimal number of clusters and classified ESCC samples into two molecular subtypes. These subtypes were identified as the high-immunity group (n=71) and low-immunity group (n=84) based on the expression profiles. The Cumulative Distribution Function (CDF) curve exhibited a smooth ascending trend, validating the rationality of the clustering. Using the “ESTIMATE” package, we calculated the stromal score, immune score, estimate score, and tumor purity for each ESCC sample. Subsequently, we employed the “ggpubr” package to illustrate the differences in tumor microenvironment scores between the high and low immunity groups through violin plots.

### Identification and enrichment analysis of significantly different IRGs

Differentially expressed genes (DEGs) were identified using “limma” R package with a cutoff value set at |log2fold change| > 0.5 and a false discovery rate (FDR) < 0.0005. The Venn diagram was drawn to screen the overlapping genes between the IRGs and DEGs. Gene Ontology (GO) enrichment and Kyoto Encyclopedia of Genes and Genomes (KEGG) analyses of the overlapping genes were performed using the “clusterProfiler” R package.

### Development and validation of prognostic features in IRGs

Using univariate Cox regression analysis, we selected IRGs with expression profiles significantly correlated with OS of ESCC patients (*P* < 0.05). Subsequently, we conducted a multivariate Cox regression analysis and constructed a risk score model:

Risk Score = Σ*i* Coef (mRNAi) * Exp (mRNAi). Here, Coef (mRNAi) represents the regression coefficient from the multivariate Cox regression for each IRG, and Exp (mRNAi) is the expression level of each IRG. Subsequently, based on the above risk score, we divided patients into high-risk and low-risk groups using the median. Kaplan-Meier analysis was employed to assess the performance of IRGs, and the TCGA-ESCC dataset was used to validate the predictive capability of this feature. Additionally, time-dependent receiver operating characteristic curves (ROC) were used to validate the prognostic prediction of the risk model by calculating the 1-year, 2-year, and 3-year Area Under Curve (AUC) values in both the training and validation sets.

### Establishment of nomogram

By conducting univariate and multivariate Cox regression analyses, we aimed to determine whether the risk score and relevant clinical parameters could serve as predictive factors associated with OS in ESCC patients. Subsequently, we utilized the R package “rms” to create a prognostic nomogram for predicting the survival of 155 ESCC patients at the time point of 1-year, 2-year, and 3-year respectively. Following this, calibration curves were plotted to assess the consistency between actual and predicted survival rates. We employed ROC curves to evaluate the performance of the risk score and relevant clinical parameters, and time-dependent ROC curves were employed to validate the predictive ability of the nomogram.

### Immunoinfiltration abundance analysis

The “CIBERSORT” method was employed separately on the transcriptional profiles of 155 cases, TCGA-ESCC data, and GSE53625 data to estimate the abundance of 22 immune cell types in ESCC samples. Samples with *P* < 0.05 were selected for subsequent analysis in the transcriptional profiles of 155 cases and GSE53625 data. Due to the limited number of samples in TCGA data, samples with *P* < 0.1 were chosen for further analysis in this dataset.

### Cell culture and treatment

HET-1A, one normal esophageal squamous epithelial cells and different ESCC cell lines TE1, TE14, TE5, TE9, KYSE150, and THP-1 were cultured with RPMI-1640 medium with 10% fetal bovine serum. THP-1 cells were induced to M0-like macrophages with 100 ng/mL phorbol ester then to M1-like macrophages with lipopolysaccharide (100ng/mL) for 48 hours or to M2-like macrophages with 20 ng/mL IL-4 for 48 hours. Small interfering RNA (siRNA) targeting SECTM1 and negative control (NC) siRNA were obtained from Ribobio Co., Ltd (Guangzhou, China). Coding sequence of SECTM1 was cloned and inserted in pcDNA3.1-HA plasmid to overexpress the gene. Transfection of siRNAs and plasmid was performed using Lipofectamine 2000 (Thermo Fisher Scientific Inc). To explore the effect of CCL5 on the course, TAK-779, a selective antagonist of CCR5, was added in the medium in the upper chambers to block the interaction between CCL5 and CCR5.

### Real-time quantitative PCR

Knockdown and overexpress efficiency of SECTM1, and the biomarker of M1 and M2 macrophages were detected using reverse transcription quantitative real-time PCR (qPCR) as our previous study (18). Briefly, total RNA was extracted using Trizol (Invitrogen Bio Inc., USA), and 2 μg RNA was used to synthesize cDNA by the PrimeScript cDNA Synthesis Kit (Takara Bio Inc., Japan). Then, SYBR Green method was used to detect the threshold cycle (Ct). Relative expression of target genes was calculated using the 2^-ΔΔCt^ formula by normalization to GAPDH expression. All experiments were done in triplicate and repeated three times at least. The siRNA sequences and primers sequences were listed in the **Supplementary Table S2**.

### Cell proliferation assay

Effect of SECTM1 on the proliferation of ESCC cells was detected using Cell Counting Kit-8 (CCK-8). Briefly, 48 hours after transfection siRNA and plasmid, ESCC cells were seeded in a 96-well plate and pre-incubated the plate for 24 hours in a humidified incubator (at 37°C, 5% CO_2_). Then 10 µL of CCK-8 solution were added to each well and the plate was incubated for 4 hours at 37°C, 5% CO_2_. Measure the absorbance at 450 nm using a microplate reader.

### Transwell assay

Cell migration and invasion abilities were determined by Transwell assay as our previous study (18). Briefly, 10 × 10^4^ cells were added into the upper chambers without Matrigel (for migration assay) or with Matrigel (for invasion assay) and 600 μl medium with 10% FBS was added into the bottom culture chamber. Then cells were cultured in an incubator with 5% CO_2_ at 37°C for 24 hr. Subsequently, non-migrated cells were removed from the inner side of the transwell and cells on the chamber bottom were fixed with 4% paraformaldehyde for 10-15 minutes and stained with crystal violet. Random five view fields were selected to take image and to count the number of transmigrated cells for each culture well. Three replicates were set for each group, and each experiment was repeated at least three times.

### Statistical analysis

The bioinformatics analyses were conducted using R (version 4.3.1). Independent t-tests were employed to assess the statistical significance of continuous variables between two groups, assuming a normal distribution of variables. For the comparison of two groups with non-normally distributed variables, the Wilcoxon test was utilized. Spearman’s test was used for correlation analysis and calculation of correlation coefficients. The significance level was set at *P* < 0.05.

## Results

### Establishing the immune subtypes of 155 patients with ESCC

**Figure 1:**
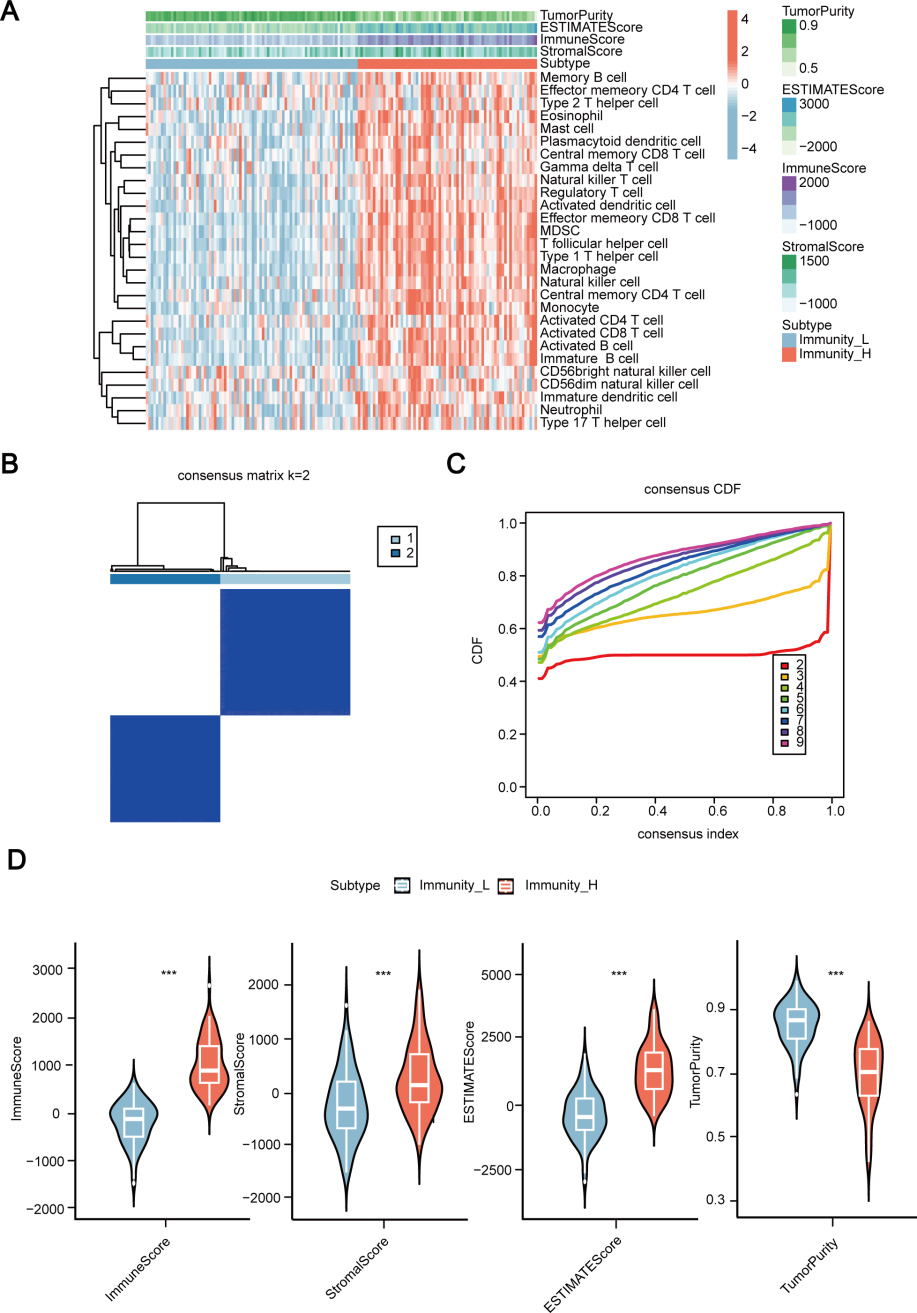
Establishment of immune subtypes in 155 cases of esophageal squamous cell carcinoma (ESCC) based on ssGSEA scores for 28 immune signals. **(A)** Clustering heatmap of 155 ESCC cases of transcriptome data incorporating ssGSEA scores. **(B, C)** Establishing two immune subtypes in 155 cases of ESCC through unsupervised clustering. **(D)** Comparison of immune scores, stromal scores, estimated scores, and tumor purity between high and low immune groups. ssGSEA, Single Sample Gene Set Enrichment Analysis; CDF, the cumulative distribution function. ESCC, Esophageal Squamous Cell Carcinoma. ***, *P* < 0.001.

First, we conducted ssGSEA analysis on the transcriptome data of 155 ESCC samples, performed unsupervised clustering on ssGSEA scores for 28 immune-related gene sets, and identified two distinct immune grouping patterns (**Figures 1A–C**). These two immune groups were referred to as the immune-high group and immune-low group (**Figure 1D**), with the immune-high group having the higher estimated score, immune score, and stromal score. It is also associated with the lower tumor purity.

### Identification of immune differential genes

**Figure 2:**
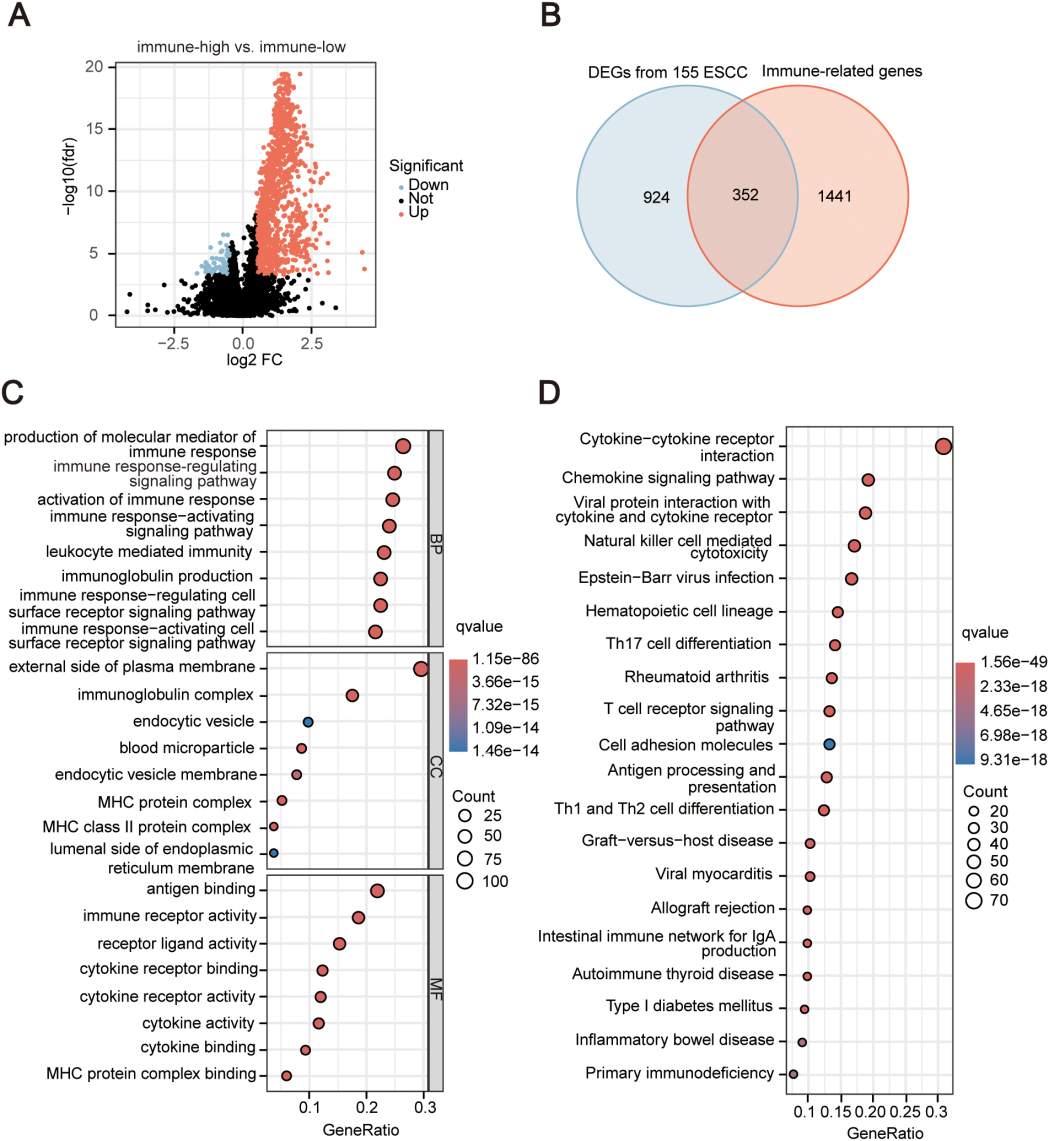
Identification and enrichment analysis of significantly differentially expressed immune-Related Genes (IRGs). **(A)** Volcano plot of differential analysis between high and low immune groups. The horizontal axis in the figure represents the log_2_-transformed differential expression fold change value (FC), and the vertical axis represents the log_10_-transformed *P*-value. The dots represent genes, and the genes that meet the criteria of FC > 1.5 and *P*-value < 0.05 are represented in red, and those that meet the criteria of FC < 0.67 and *P*-value < 0.05 are represented in blue. Non-significant differential metabolites are represented in black. **(B)** Venn diagram depicting the intersection of differentially expressed genes and immune-related genes. **(C)** Bubble plot of Gene Ontology (GO) analysis for immune-related DEGs. BP, Biological Process; CC, Cellular Component; MF, molecular function. The horizontal axis in the figure represents the significant pathways, and the vertical axis represents the gene ratios. The size of the circle represents the number of genes, while the color represents the q value. **(D)** Bubble plot of Kyoto Encyclopedia of Genes and Genomes (KEGG) analysis for immune-related differentially expressed genes. The horizontal axis in the figure represents the significant pathways, and the vertical axis represents the gene ratios. The size of the circle represents the number of genes, while the color represents the q value.

Comparing the differences in gene expression levels between samples from the high immune group and the low immune group (immune-high *vs.* immune-low), we identified a total of 1276 DEGs, including 1188 upregulated genes and 88 downregulated genes (**Figure 2A**). A collection of 2483 immune-related genes (IRGs) was obtained from IMMPORT, but only 1793 IRGs were expressed in 155 ESCC data, and the intersection between the DEGs and the IRGs resulted in 352 immune-related DEGs (**Figure 2B**). We further explored the biological functions and pathways of the 352 IRGs. GO enrichment showed that these IRGs are associated with the positive regulation of various immune cell activations and immune receptor signaling pathways (**Figure 2C**). KEGG results reveal a strong correlation between differential immune genes and the pathway of interactions of cytokines and their receptors, natural killer (NK) cell-mediated cytotoxicity, and differentiation of Th1 and Th2 cells, among various immune signaling pathways (**Figure 2D**). The detailed results of enrichment were list in **Supplementary Tables S3**, **S4**.

### Development of prognostic characteristics of IRGs

**Figure 3:**
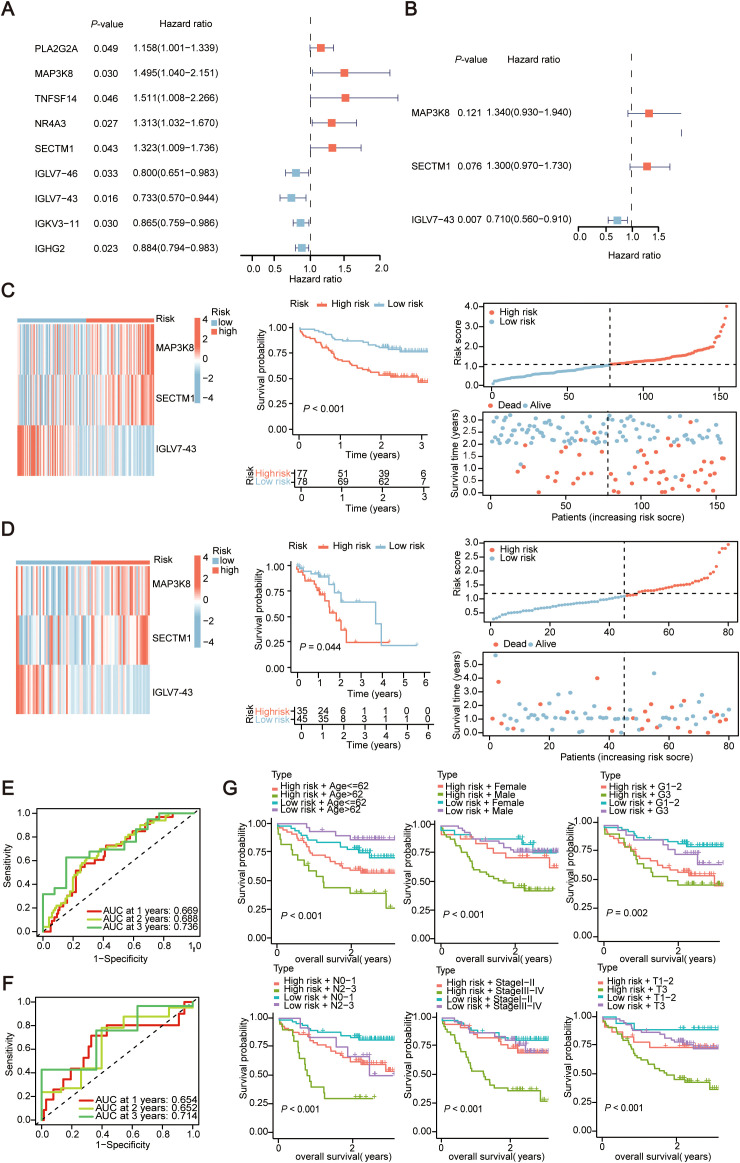
Evaluate the prognostic characteristics of Immune-Related Genes (IRGs) constructed in 155 cases of esophageal squamous cell carcinoma data. **(A)** Univariate Cox regression forest plot of the 9 candidate genes. **(B)** Multivariate Cox regression forest plot of the three final selected genes (MAP3K8, SECTM1, IGLV7-43). **(C, D)** Heatmap depicting the expression of three genes between high and low-risk score groups and corresponding Kaplan-Meier survival curves, risk score curve graph and scatter plot of risk scores in the transcriptional data of 155 ESCC cases and TCGA ESCC cohort. The dashed line represents the distribution of individual risk scores, categorizing patients into low-risk and high-risk groups. In scatter plot of risk scores, red points denote deceased patients, while green points represent survivors. With an increase in risk scores, more patients experience mortality. **(E, F)** Time-dependent ROC curves for the training set (155 ESCC cohort) and Validation set (TCGA ESCC cohort). AUC, area under curve; ROC, receiver operating characteristic curve. **(G)** Kaplan-Meier survival curves for risk score groups combined with age, gender, and G-stage in the transcriptional data of 155 ESCC cases. The horizontal axis in the figure represents overall survival time, and the vertical axis represents the cumulative survival rate. Colors represent different groups. TCGA, The Cancer Genome Atlas Program; ESCC, Esophageal Squamous Cell Carcinoma.

Univariate Cox regression analysis was conducted on the above 352 IRGs. The results showed that there were nine IRGs associated with the OS of ESCC patients significantly (*P* < 0.05) (**Figure 3A**). Subsequently, multivariate Cox analysis was performed on these 9 immune prognostic genes, and through stepwise regression, 3 genes (MAP3K8, SECTM1, IGLV7-43) were selected as prognostically relevant genes to establish a prognostic model (**Figure 3B**). Based on these three genes, a prognostic model was constructed. The risk score was calculated by multiplying the gene expression level by the coefficient obtained from the multivariate Cox regression, i.e., riskscore = (0.295 × expression of MAP3K8) + (0.262 × expression of SECTM1) + (-0.355 × expression of IGLV7-43). According to the median risk score, 155 ESCC patients were divided into high-risk and low-risk groups. A heatmap was generated to illustrate the RNA expression level of the three genes in the two groups (**Figure 3C**). KM curves, survival curves, and survival status plots all showed that ESCC patients in high-risk group had poorer prognosis compared with the patients in low-risk group (**Figure 3C**). To validate the effectiveness of the three genes (MAP3K8, SECTM1, IGLV7-43) in predicting prognosis within the model, we calculated the risk scores for 80 ESCC sequencing data from TCGA using the same formula, with the median risk score from the training set of 155 transcriptome data as the baseline. The TCGA ESCC data showed the same trend (**Figure 3D**). Time-dependent ROC curves showed that the AUC values of 1-year, 2-year, and 3-year survival rates in the training set of 155 ESCC samples were 0.669, 0.688, and 0.736, respectively; in the validation set of TCGA ESCC, the AUC values of 1-year, 2-year, and 3-year survival rates were 0.654, 0.652, and 0.714, respectively (**Figures 3E, F**).

### Establishment of nomogram

**Figure 4:**
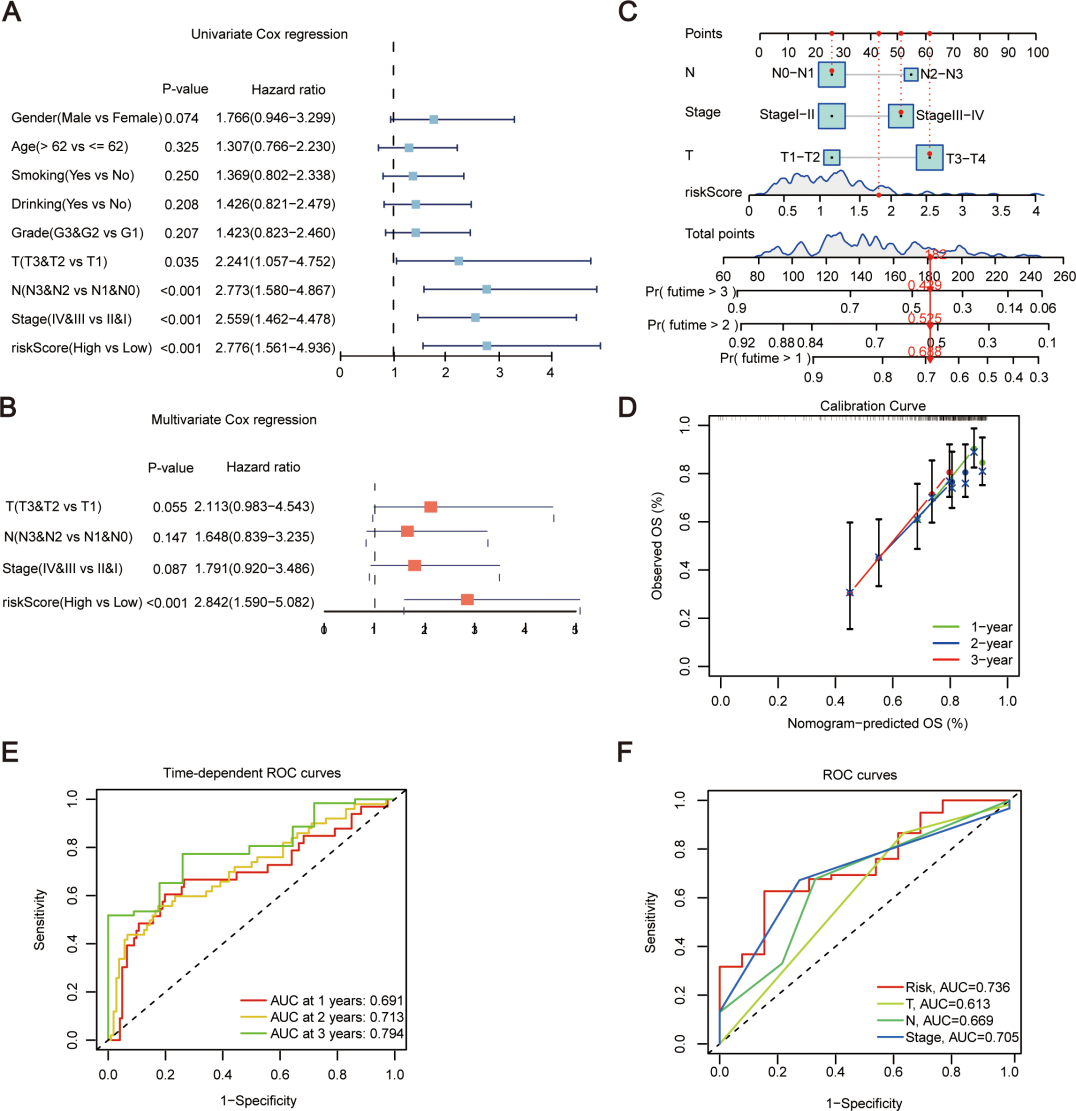
Establishment of nomogram. **(A)** Forest plot of Univariate Cox regression analysis, incorporating some clinical traits and riskScore. **(B)** Forest plot of Multivariate Cox regression analysis for traits showing significance in single-factor Cox analysis. **(C)** Establishment of Nomogram incorporating N, Stage, T, and riskScore features. **(D)** Evaluation of Nomogram results using calibration curves. **(E)** Evaluation of Nomogram results using time-dependent ROC curves. **(F)** ROC curves for risk scores and other relevant clinical parameters. T, pathologic tumor stage; N, pathologic lymph nodes stage; OS, overall survival; ROC, receiver operating characteristic curve.

Then gender, age, smoking status, alcohol consumption, G stage, T stage, N stage, clinical stage, and riskScore were included as factors for Univariate Cox regression analyses. The analyses indicated that T stage, N stage, stage, and riskScore were significant (*P* < 0.05) (**Figure 4A**). These clinical features were then subjected to multivariate Cox regression (**Figure 4B**). Subsequently, a prognostic nomogram was constructed based on the above variables (**Figure 4C**). Simultaneously, we used calibration curves (**Figure 4D**) and time-dependent ROC curves (**Figure 4E**) to evaluate the specificity and sensitivity of this result, and according to the generated data, the AUC values for 1-year, 2-year, and 3-year survival rates were 0.691, 0.713, and 0.794, respectively. Finally, ROC curve results demonstrated that riskScore had a better predictive ability compared to other relevant clinical parameters (**Figure 4F**). And the risk score combined with other clinical parameters can also effectively distinguish the clinical prognosis of patients (**Figure 3G**).

### SECTM1 acts as the primary gene in the model

**Figure 5:**
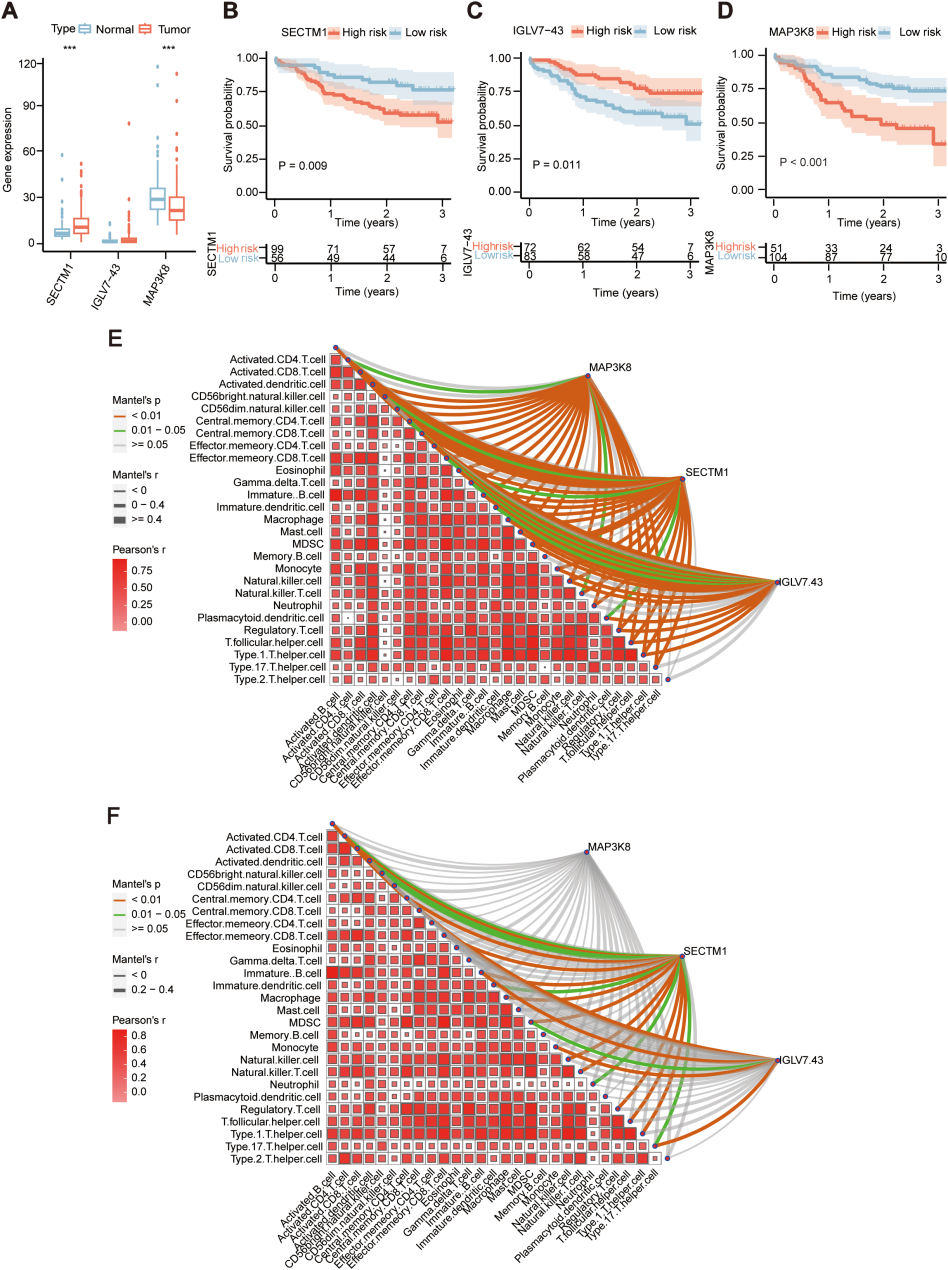
Expression and prognosis of the 3 genes in ESCC tissues in the prognostic model. **(A)** Comparison of the expression of the three genes in 155 ESCC tumor samples and adjacent normal samples in the prognostic model. ***, P < 0.001 **(B)** Prognostic differences in the high and low expression groups of MAP3K8 in 155 ESCC samples. **(C)** Prognostic differences in the high and low expression groups of SECTM1 in 155 ESCC samples. **(D)** Prognostic differences in the high and low expression groups of IGLV7-43 in 155 ESCC samples. **(E)** Correlation of the three genes in the prognostic model with immune signals in 155 ESCC samples. **(F)** Correlation of the three genes in the prognostic model with immune signals in TCGA-ESCC cohort. MAP3K8, Mitogen-Activated Protein Kinase Kinase Kinase 8; IGLV7-43, Immunoglobulin Lambda Variable 7-43; SECTM1, Secreted And Transmembrane 1; TCGA, The Cancer Genome Atlas Program; ESCC, Esophageal Squamous Cell Carcinoma.

During the examination of the transcriptional data from 155 ESCC samples, an assessment was conducted on the three genes (MAP3K8, SECTM1, IGLV7-43) within the prognostic model, it was found that MAP3K8 was significantly higher in adjacent normal tissues than in tumor tissues, while SECTM1 was significantly higher in tumor tissues than in adjacent normal tissues (**Figure 5A**). Analysis of the impact of these three genes on prognosis revealed that high expression of MAP3K8 and SECTM1 was associated with poorer prognosis, while low expression of IGLV7-43 was linked to poorer prognosis (**Figures 5B–D**). Further examination of the correlation between the three genes of the prognostic model and immune signals in the transcriptional data of 155 samples revealed that SECTM1 had the highest correlation with immune signals (**Figure 5E**). And TCGA ESCC cohort also showed similar results (**Figure 5F**). From TCGA data, we also found SECTM1was higher in tumor tissues than that in normal tissues in in various types of cancer (**Supplementary Figure S1**). And high SECTM1 was associated with poorer prognosis in TCGA ESCC samples (**Supplementary Figure S2**). Therefore, SECTM1 was chosen for subsequent studies.

### SECTM1 promoted the malignant phenotypes of ESCC cell

**Figure 6:**
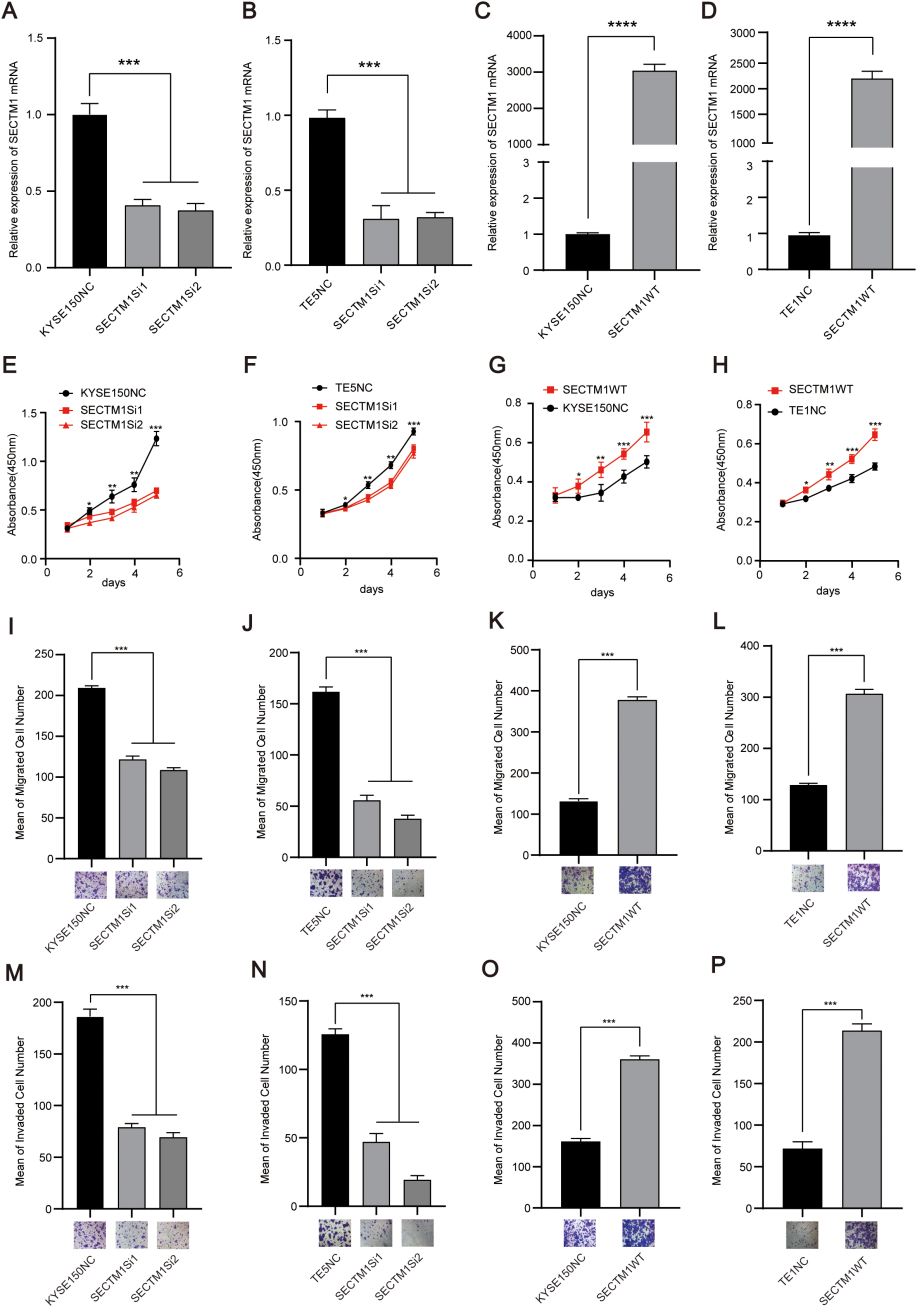
SECTM1 promotes the proliferation, migration and invasion of ESCC cells. **(A, B)** Knockdown efficiency of SECTM1 in KYSE150 cells and TE5 cell. **(C, D)** Overexpression efficiency of SECTM1 in KYSE150 cells and TE1 cells. **(E, F)** Effect of SECTM1 knockdown on the proliferation ability in KYSE150 cells and TE5 cells. **(G, H)** Effect of SECTM1 overexpression on the proliferation ability in KYSE150 cells and TE1 cells. **(I, J)** Effect of SECTM1 knockdown on the migration ability in KYSE150 cells andTE5 cells. **(K, L)** Effect of SECTM1 overexpression on the migration ability in KYSE150 cells and TE1 cells. **(M, N)** Effect of SECTM1 knockdown on the invasion ability in KYSE150 cells and TE5 cells. **(O, P)** Effect of SECTM1 overexpression on the invasion ability in KYSE150 cells and TE1 cells. *, P < 0.05; **, P < 0.01; ***, P < 0.001; ****.P <0.0001. Bar = 100μm. SECTM1, Secreted And Transmembrane 1; ESCC, Esophageal Squamous Cell Carcinoma.

We detected the endogenous expression of SECTM1 in different ESCC cell lines. The results showed that SECTM1 was expressed at the highest level in TE14, TE5, and TE9, moderate inKYSE150 and lower in TE1 (**Supplementary Figure S3**). After observing the cell states, we chose KYSE150 and TE5 cells for SECTM1 knockdown and TE1 for overexpression. As the KYSE150 cell exhibited a relatively favorable state, it was selected for both knockdown and overexpression. To investigate the function of SECTM1 in ESCC cell lines, we transfected siRNA into the selected cell lines for knockdown and SECTM1 overexpression plasmids into the selected overexpressing cell lines. RNA was extracted and RT-qPCR was performed between 48-72 hours to verify the knockdown and overexpression efficiency. The experimental results showed that siRNA successfully reduced the expression level of SECTM1 in cells (**Figures 6A, B**), while the SECTM1 overexpression plasmid effectively increased the expression level of SECTM1 (**Figures 6C, D**). The CCK-8 experiment revealed that knockdown of SECTM1 significantly inhibited the proliferation of KYSE150 and TE5 cells (**Figures 6E, F**). In KYSE150 and TE1 cell lines, overexpression of SECTM1 significantly promoted cell proliferation (**Figures 6G, H**). In KYSE150 and TE5 cell lines, knockdown of SECTM1 significantly inhibited the migration of KYSE150 and TE5 cells, as observed in the transwell migration experiment (**Figures 6I, J**). In KYSE150 and TE1 cell lines, overexpression of SECTM1 significantly promoted the migration of KYSE150 and TE1 cells (**Figures 6K, L**). Furthermore, the knockdown of SECTM1 significantly inhibited the invasion of KYSE150 and TE5 cells (**Figures 6M, N**). In KYSE150 and TE1 cell lines, overexpression of SECTM1 promoted the invasion of KYSE150 and TE1 cells, as demonstrated in the invasion experiment (**Figures 6O, P**). These results suggest that SECTM1 may play a pro-cancer role in ESCC, consistent with clinical prognostic analysis.

### The correlation between SECTM1 and immune signal infiltration in the tumor microenvironment

**Figure 7:**
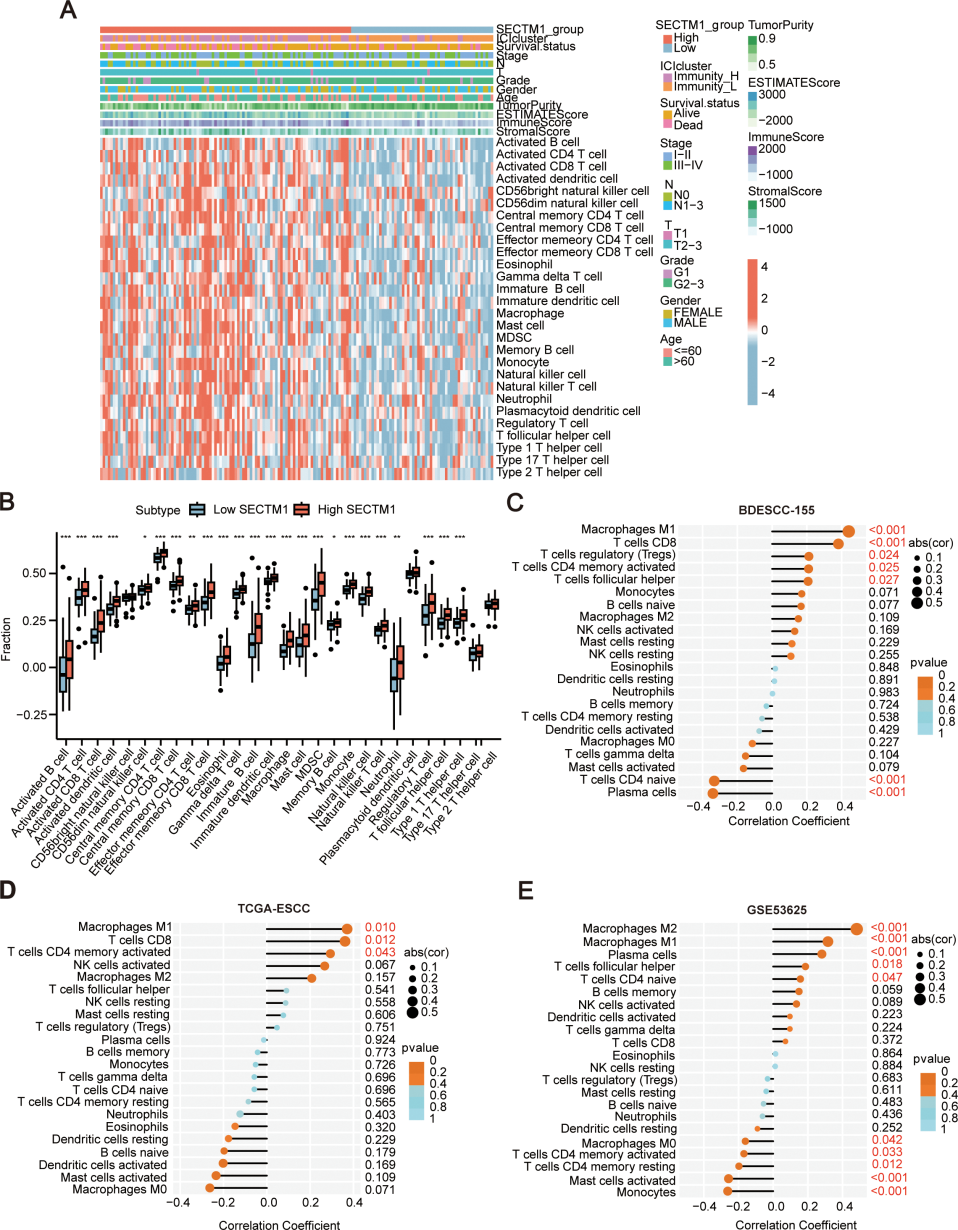
The correlation between SECTM1 and immune signal infiltration in the tumor microenvironment in ESCC. **(A)** Heatmap of ssGSEA scores for 155 ESCC cases transcriptome data (with a focus on SECTM1 high-low expression). **(B)** Boxplot depicting the differences in immune signals between SECTM1 high and low expression groups in 155 ESCC cohort. * represents significant differences. *, P < 0.05; **, P < 0.01; ***, P < 0.001. **(C–E)** The correlation between SECTM1 and the abundance of 22 immune cell infiltrations in 155 ESCC cohort, TCGA ESCC cohort and GSE53625. SECTM1, Secreted And Transmembrane 1; TCGA, The Cancer Genome Atlas Program; ESCC, Esophageal Squamous Cell Carcinoma; ssGSEA, Single Sample Gene Set Enrichment Analysis.

TME significantly influences malignant phenotypes of tumor cells (5). Thus, through the analysis of the transcriptome data of 155 ESCC samples, we further explored the correlation between SECTM1 expression and immune signals. Based on the optimal threshold point of the ROC curve for SECTM1 expression, the samples were divided into SECTM1 high and low expression groups, and the changes in immune signals in both groups were observed. The results revealed that the high-expression of SECTM1 was mainly positively correlated with immune signals such as macrophages, NK cells, and T cells (**Figure 7A**). Analyzing the differences in immune signals between the SECTM1 high and low expression groups, it was found that all immune signals in the SECTM1 high-expression group were higher than those in the low-expression group (**Figure 7B**). These results collectively suggest that SECTM1 may be positively correlated with immune infiltration.

Furthermore, the correlation between SECTM1 and different immune cell infiltration abundance was analyzed using the “CIBERSORT” algorithm in 155 ESCC data, GSE53625, and TCGA-ESCC databases. The results revealed that SECTM1 is associated with macrophages, CD8+ T cells, and NK cells. Additionally, SECTM1 showed a positive correlation with M1 and M2 macrophages and a negative correlation with M0 macrophages (**Figures 7C–E**). The three datasets exhibited similar trends.

### SECTM1 promoted macrophage polarization towards the M2-like phenotype

**Figure 8:**
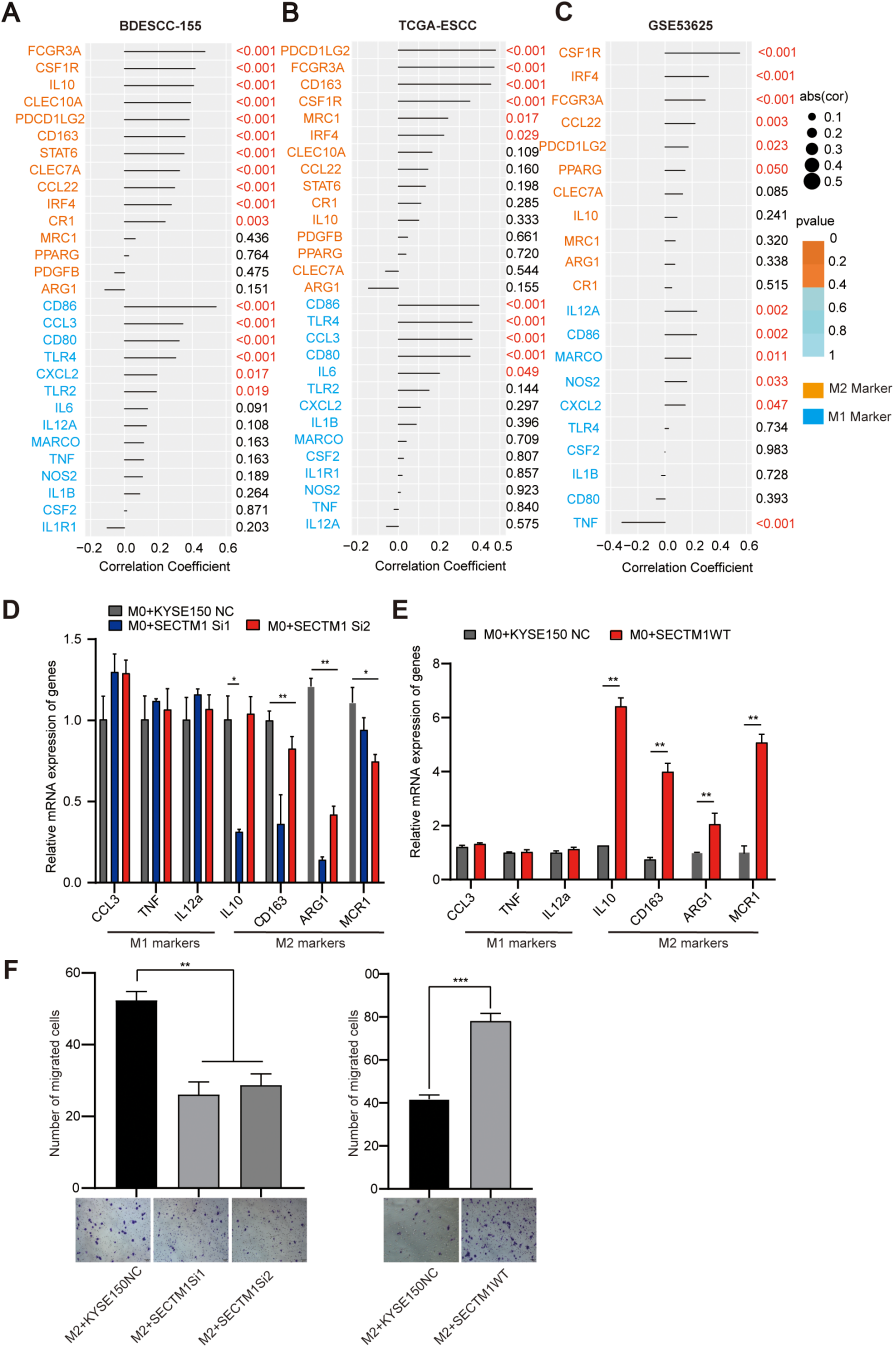
The impact of SECTM1 on macrophage phenotype. **(A-C)** Correlation between SECTM1 and M1, M2 markers in 155 ESCC transcriptome data, TCGA-ESCC data and GSE53625 data. **(D, E)** The impact of SECTM1 knockdown and SECTM1 overexpression in KYSE150 cells on the expression of polarization markers in M0 macrophages. **(F)** The effect of SECTM1 overexpression and knockdown in KYSE150 cells on the migratory ability of M2 macrophages. *, *P* < 0.05; **, *P* < 0.01; ***, *P* < 0.001. Bar = 100μm. SECTM1, Secreted And Transmembrane 1; TCGA, The Cancer Genome Atlas Program; ESCC, Esophageal Squamous Cell Carcinoma; M0, M0-like macrophages; M1, M1-like macrophages; M2, M2-like macrophages.

Since M1 and M2 macrophages have significantly different biological functions, further analysis of the correlation between SECTM1 and the main markers of M1 and M2 in 155 cases, TCGA, and GSE53625 data reveals that the positive correlation of SECTM1 with M2 markers may be greater than its correlation with M1 markers (**Figures 8A–C**).

We further collected the supernatants from KYSE150 cells with knocked down and overexpressed SECTM1 as conditioned medium (CM), to treat M0-like cells induced from THP-1 cells. Subsequently, qPCR was conducted to detect alterations in the surface markers of M1 and M2 macrophages. The results showed that compared to the control group, the CM from KYSE150 with knocked down SECTM1 significantly reduced the surface markers of M2 macrophages, such as MCR1, CD163, and ARG1, with a slightly upward trend in the expression of M1 macrophage markers, which was not very pronounced (**Figure 8D**). On the contrary, treatment of macrophages with CM overexpressing SECTM1 significantly upregulated the expression of surface markers for M2 macrophages, such as IL-10, CD163, ARG1, and MCR1, with no significant trend observed for M1 macrophage markers (**Figure 8E**).

Furthermore, a transwell co-culture experiment was conducted to study the chemotactic effect of SECTM1 on M2 macrophages. The results showed that the CM from SECTM1-knocked down cells significantly inhibited the migration of M2 macrophages compared to the control group (**Figures 8A, B**). Conversely, the CM with overexpressed SECTM1 significantly promoted the migration of M2 macrophages (**Figure 8F**). Integrating the findings from bioinformatics analysis and cell experimental results, we elucidated that overexpression of SECTM1 may promote the polarization of macrophages from M0 to M2 and promote M2 macrophages chemotaxis to tumor cells.

### SECTM1 may exert a pro-cancer effect through CCL5

**Figure 9:**
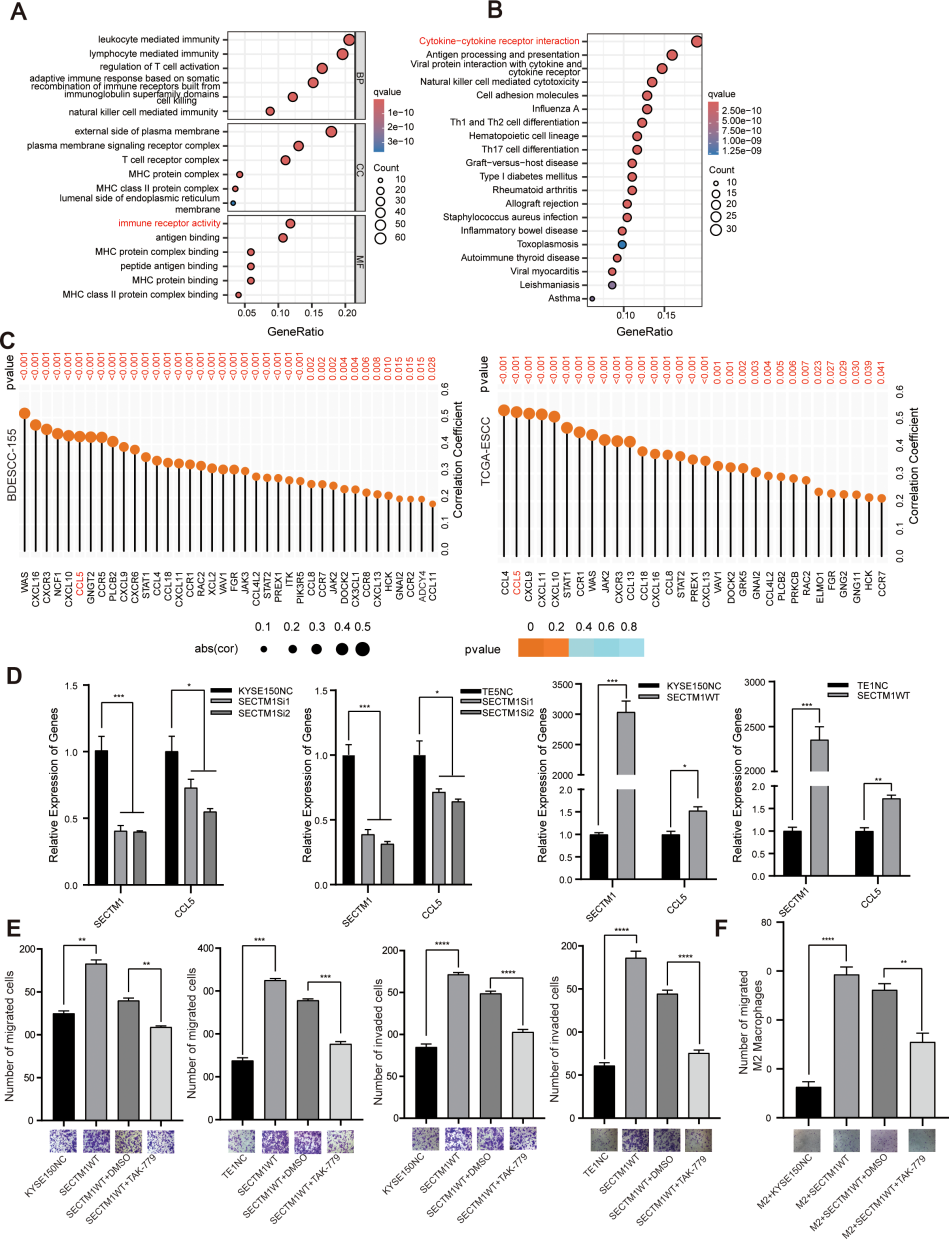
CCL5 is a potential mediator of SECTM1’s pro-cancer effect. **(A, B)** GO and KEGG analysis on DEGs based on the analysis of single-gene bioinformatics on SECTM1 in the transcriptome data from 155 ESCC cases. The horizontal axis in the figure represents the significant pathways, and the vertical axis represents the gene ratios. The size of the circle represents the number of genes, while the color represents the q value. **(C)** Correlation between SECTM1 and chemokines in 155 ESCC cohort and TCGA-ESCC cohort. The size of the circle represents the correlation coefficient, while the color represents the P value. **(D)** The impact of SECTM1 knockdown and overexpression on CCL5 mRNA expression in ESCC cells. **(E)** The impact of CCL5 inhibitor TAK-799 on the migration and invasion of ESCC cells overexpressing SECTM1. **(F)** The impact of CCL5 inhibitor TAK-799 inhibited the migration ability of M2 macrophages treated with conditioned medium from ESCC cells overexpressing SECTM1. *, *P* < 0.05; **, *P* < 0.01; ***, *P* < 0.001, ****, *P* < 0.0001. Bar = 100μm. CCL5, C-C Motif Chemokine Ligand 5; SECTM1, Secreted And Transmembrane 1; TCGA, The Cancer Genome Atlas Program; ESCC, Esophageal Squamous Cell Carcinoma; M1, M1-like macrophages; M2, M2-like macrophages.

The interaction between tumor cells and immune cells primarily depends on the interplay of various chemotactic factors, cytokines, and their receptors. Differential analysis was performed on all genes between the SECTM1 high and low expression groups, and the differentially expressed genes were selected for GO and KEGG analysis. It was found that these genes were mainly enriched in immune-related pathways such as cytokine-cytokine receptor interaction, Th cell differentiation, and natural killer cell-mediated cytotoxicity (**Figures 9A, B**). Both GO and KEGG results suggested that SECTM1 might be highly associated with chemotactic factor pathways. We then explored the correlation between SECTM1 and various chemotactic factors in the transcriptome data of 155 ESCC cases and TCGA-ESCC data. The analysis revealed a strong correlation between SECTM1 and chemotactic factors and CCL5 was selected as the main chemotactic factor for further study (**Figure 9C**).

Further experiments indicated that SECTM1 knockdown in KYSE150 and TE5 cells resulted in a significant decrease in the expression of CCL5. Conversely, overexpression of SECTM1 in KYSE150 and TE1 cells resulted in a significant increase in CCL5 expression (**Figure 9D**). In ESCC cells overexpressing SECTM1, the addition of TAK-779, a selective antagonist of CCR5, significantly reversed the migration and invasion induced by high SECTM1 expression (**Figure 9E**). Meanwhile, TAK-779 significantly reversed the migration of M2 macrophages induced by CM with high SECTM1 expression (**Figure 9F**). These results suggest that SECTM1 may exert its pro-cancer effect by inducing the expression of the chemotactic factor CCL5.

## Discussion

TME is believed to contribute to immune evasion, suppression of apoptosis, and promotion of proliferation, angiogenesis, invasion, and metastasis of cancer cells, playing an essential role in the development and progression of tumors. Therefore, studying immune infiltration is essential for understanding the relationship between tumors and the immune system (19). ESCC is a common malignant tumor of the digestive tract, and studies suggest its close association with the tumor microenvironment (20, 21). The microenvironment of ESCC involves complex interaction networks between various cells and molecules. In ESCC microenvironment, inflammation and immune cells play a significant role, promoting cancer development, invasion, and metastasis.

In this study, we utilized ssGSEA (22–26) to classify two ESCC subtypes— immune-high and immune-low—based on the transcriptome data of 155 ESCC cases. Immune-high patients had significantly higher immune scores, indicating higher immune activity. We identified 352 immune-related genes (IRGs) between the two ESCC subtypes, which were cross-validated using IRGs from the IMMPORT database. From this analysis, we pinpointed nine immune-related prognostic genes, ultimately narrowing it down to three: MAP3K8, SECTM1, and IGLV7.43. These genes formed a prognostic model that was validated using data from the TCGA-ESCC cohort.

Among these three genes, IGLV7-43 is a lambda-type immunoglobulin light chain, known for its higher frequency in ABO-incompatible kidney transplant patients, suggesting its involvement in specific immune responses (27). MAP3K8 is a serine/threonine kinase associated with various immunosuppressive checkpoints, chemokines, and receptors, indicating its potential role in tumor immunity (28). Furthermore, research reports indicate that MAP3K8 plays a central role in regulating lung inflammation and Cox-2-mediated PGE2 production. The decreased expression of MAP3K8 may be a contributing factor to the development of pulmonary fibrosis (29). In clear cell renal cell carcinoma (CCRC), the overexpression of MAP3K8 correlates with poor survival and may be involved in in cancer-related inflammation through NF-κB and Toll-like receptor signaling pathways (30). SECTM1 exhibited the highest correlation with immune signals among the three genes in the present study. It is an IFN-γ-inducible molecule broadly expressed and recognized as a ligand for CD7 (13). SECTM1 enhanced the proliferation of activated T cells synergizing with anti-CD28 (31). SECTM1 CAR-T cells have demonstrated effectiveness against malignant cells with CD7-high expression, both *in vitro* and *in vivo* (32). Additionally, low SECTM1 has shown potential in suppressing glioblastoma, indicating its promise as a biomarker and therapeutic target for this malignancy (33). However, its functional role in ESCC and its correlation with tumor immunity remain unexplored.

To further explore SECTM1’s biological role in ESCC, we conducted preliminary experiments. Our findings indicated that SECTM1 overexpression significantly promoted cell migration, invasion, and proliferation in ESCC cells, while its knockdown inhibited these processes. Bioinformatics analysis revealed that SECTM1’s downstream genes were enriched in immune-related pathways such as cytokine-cytokine receptor interaction, Th1 and Th2 cell differentiation, and natural killer cell-mediated cytotoxicity. This suggests that SECTM1’s pro-cancer effect in ESCC may be related to the immune mechanism. Further analysis indicated there was a significant correlation between SECTM1 and macrophages infiltration. Previous studies indicate that M1 macrophages are mostly associated with anti-tumor effects, while M2 macrophages are mostly associated with pro-tumor activities. Our analysis showed a stronger correlation between SECTM1 and M2 macrophage markers. Further experimental results demonstrated that conditioned media from SECTM1 knockdown cells inhibited the migration of both M1 and M2 macrophages, although the effect was more pronounced on M2 macrophages. Conversely, conditioned media from SECTM1-overexpressing cells significantly enhanced the migration of both macrophage types, again with a greater impact on M2 macrophages. Meanwhile overexpressed SECTM1 may promote M0 macrophages towards M2 polarization and enhance M2 infiltration into tumors. Integrating bioinformatics and molecular biology experiments, our study highlights the prominent role of SECTM1 in regulating M2-type macrophages within the tumor microenvironment of ESCC.

In conclusion, a novel prognostic prediction model based on three IRGs (MAP3K8, SECTM1, IGLV7-43) was developed, which effectively predicts the survival rates of ESCC patients. Further results indicated SECTM1 plays a pro-tumorigenic role via promoting the malignant phenotypes of ESCC cells and M2 polarization of macrophages, which was correlated with chemokine signaling pathways, particularly with CCL5.

## Data Availability

The original contributions presented in the study are included in the article/**Supplementary Material**. Further inquiries can be directed to the corresponding authors.

## Supplementary material

Supplementary Figure 1 Differences of SECTM1 expression between cancer tissue and normal tissue in different types of cancer from TCGA dataset. The horizontal axis in the figure represents different types of tumor, and the vertical axis represents the expression of SECTM1 mRNA. Red means tumor tissues and blue represents normal tissues. TCGA, The Cancer Genome Atlas Program; *, *P* < 0.05; **, *P* < 0.01; ***, *P* < 0.001.

Supplementary Figure 2 Prognostic differences between the high and low expression groups of SECTM1 in TCGA ESCC cohort. The horizontal axis in the figure represents overall survival time, and the vertical axis represents the cumulative survival rate.

Supplementary Figure 3 Expression of SECTM1 in different ESCC cell lines.
