# Supplementary figures and images for: SECTM1 acts as an immune-related biomarker of poor prognosis and promotes cancer progression by modulating M2 macrophage polarization in esophageal squamous cell carcinoma
Source: Front Immunol. 2025 Jan 29;16:1507227. doi: 10.3389/fimmu.2025.1507227 (PMC11814170; doi:10.3389/fimmu.2025.1507227)

Figure S1

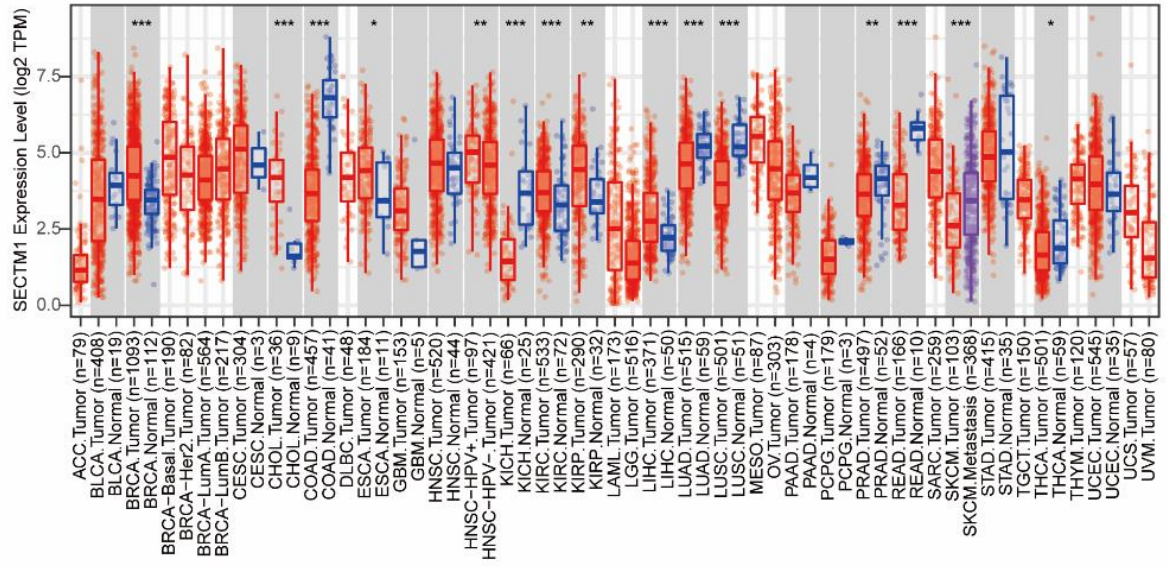

Figure S2

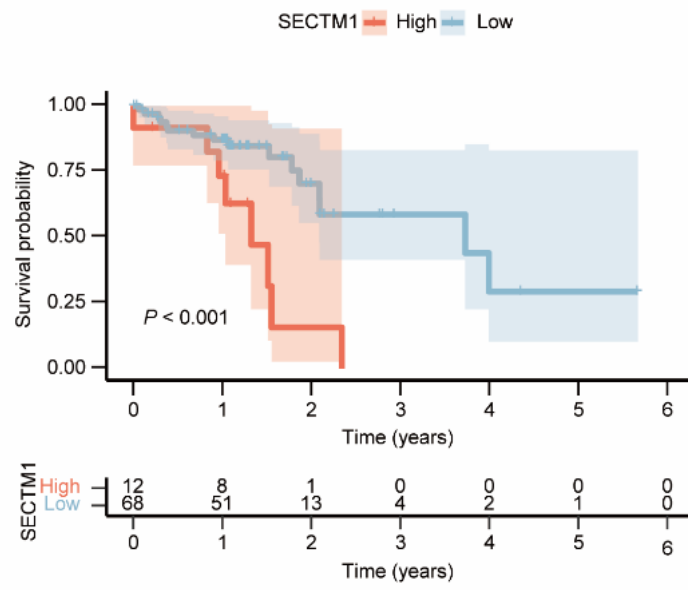

Figure S3

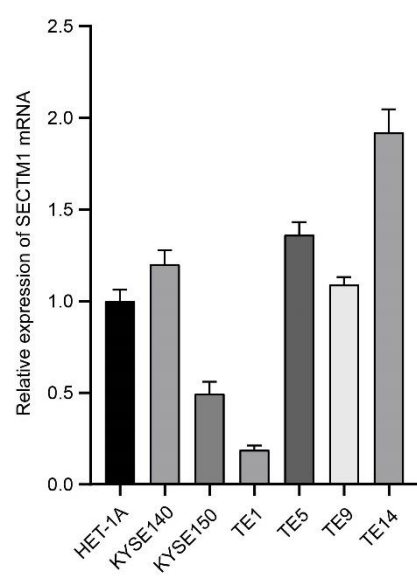

Supplement: Supplementary Figure 1 — Differences of SECTM1 expression between cancer tissue and normal tissue in different types of cancer from TCGA dataset. The horizontal axis in the figure represents different types of tumor, and the vertical axis represents the expression of SECTM1 mRNA. Red means tumor tissues and blue represents normal tissues. TCGA, The Cancer Genome Atlas Program; *, P < 0.05; **, P < 0.01; ***, P < 0.001. [file DataSheet1.pdf]
